# Perspective for CAR T-Cell Therapy in Underrepresented Populations: A Hypothesis-Generating CD19 Genomic Analysis

**DOI:** 10.3390/jpm16070343

**Published:** 2026-06-25

**Authors:** Maysa Al-Hussaini, Anas Al Okaily, Osama Alsmadi

**Affiliations:** Department of Cell Therapy and Applied Genomics, King Hussein Cancer Center, Amman 11941, Jordan; mhussaini@khcc.jo (M.A.-H.); aa.12682@khcc.jo (A.A.O.)

**Keywords:** CAR T-cell therapy, CD19, pharmacogenomics, population genetics, precision medicine, Middle East, health disparities, consanguinity, founder effect, antigen escape

## Abstract

CD19-directed chimeric antigen receptor (CAR) T-cell therapy has fundamentally transformed the treatment landscape for relapsed and refractory B-cell malignancies, yet antigen escape remains a persistent therapeutic challenge that limits long-term remission durability. While antigen loss is typically considered a somatic event acquired during tumor evolution under therapeutic selective pressure, germline CD19 polymorphisms could theoretically influence CAR-binding kinetics, alter epitope presentation, and modulate therapeutic outcomes in ways that remain largely not characterized. Unfortunately, Middle Eastern populations are underrepresented in pharmacogenomic databases and CAR-T clinical trials, creating a knowledge gap that may perpetuate global health disparities in access to precision immunotherapy. We analyzed publicly available whole-exome sequencing data from 1196 individuals of Arab origin to comprehensively characterize CD19 variants with potential relevance to CAR T-cell immunotherapy. The L174V (rs2904880) variant stood out, and showed the Valine/Valine (V/V) genotype frequency was 65.3%, corresponding to a V174 allelic frequency of 76.6%, while the minor allele, L174, has a frequency of 23.4%. The missense mutation (c.520C > G) responsible for this variant results in a leucine-to-valine (L174V) substitution at position 174 of the CD19 protein, relative to the reference genome. The cohort genotypes (CC, CG, and GG) exhibited a significant deviation from Hardy–Weinberg equilibrium (*p* < 0.00001). While this deviation is consistent with the high consanguinity rates (25–60%) amongst Arab populations, it remains not fully explained, and may be attributed to population structure, relatedness, or technical factors. We further emphasize that our computational analysis cannot establish any direct clinical or functional impact due to this variant, and therefore we refrain from suggesting any specific actions at the current time. In light of these findings, we hypothesize that the distinctive genetic architecture of consanguineous populations should not be viewed as a confounding variable. Instead, it presents a unique opportunity to investigate the clinical relevance of germline variation in the context of precision oncology, particularly at therapy-relevant loci, pending functional validation.

## 1. Introduction

Chimeric Antigen Receptor T-cell (CAR-T) therapy is a transformative advance in oncology, offering durable remissions for relapsed/refractory B-cell malignancies [1]. Second-generation CD19-targeting CARs (tisagenlecleucel, axicabtagene ciloleucel) have demonstrated remarkable efficacy in B-ALL and DLBCL [2,3]. They achieve 80–90% complete remission rates in pediatric/young adult B-ALL [4].

Mechanistically, autologous T cells are harvested and genetically modified to express a chimeric receptor with an extracellular scFv recognizing CD19, a CD3ζ signaling domain, and costimulatory domains (CD28 or 4-1BB) that enhance persistence [5]. Upon reinfusion, they proliferate exponentially and kill CD19+ B cells via perforin/granzyme and cytokine pathways [6].

Despite these successes, 30–50% of patients relapse, with CD19 modulation accounting for many resistance cases [7]. Antigen escape occurs via multiple mechanisms: transcriptional downregulation, alternative splicing, lineage switching, and, most relevant here, missense mutations disrupting the CAR binding epitope [8,9].

Most FDA-approved CAR-T products use the FMC63 scFv, which recognizes a specific epitope within the CD19 extracellular domain [10]. Single amino acid substitutions at this interface can reduce or abolish CAR-T recognition, favoring tumor clones with such mutations. Clinical studies have documented acquired somatic CD19 mutations in patients relapsing after FMC63-based therapy, including L174 mutations that impair binding [11,12].

### 1.1. The L174V Polymorphism: A Germline Variant in the CAR Binding Domain

The L174V variant (rs2904880) is a leucine-to-valine substitution at position 174 of CD19, within the minimal recognition domain of the FMC63 scFv [10]. Structural and functional studies suggest it may influence binding kinetics, but clinical significance remains undefined [13,14].

While somatic L174 mutations are acquired escape mutations post-CAR-T, the L174V variant also exists as a germline polymorphism in the general population [15]. Germline variants are present from birth and may affect CD19 structure/density across all B cells, whereas somatic mutations arise in malignant clones under selective pressure. The clinical significance of germline L174V for CAR-T outcomes remains unexplored, representing today a critical knowledge gap.

Middle Eastern populations are underrepresented in major genomic databases and CAR-T trials. In gnomAD v4.1, Middle Eastern individuals constitute <1% of samples despite representing ~5% of the global population [16]. This disparity is even more pronounced in pharmacogenomic studies examining therapy-relevant loci, where Arab individuals are frequently absent.

This gap matters due to unique demographics: Arab populations have high consanguinity rates (25–60%) [17], leading to pronounced founder effects where specific variants become concentrated [17]. The Qatar Genome Program for example found that 62.5% of Qatari participants carry at least one recessive pathogenic variant, with homozygosity 5.8-fold higher than in non-Arab ancestries [18]. Thus, variants rare in Europeans may be common and clinically significant in Arab populations, and vice versa.

### 1.2. Hypothesis and Study Rationale

We hypothesize that Arab populations, with high consanguinity and founder effects, may exhibit distinct allele frequencies and homozygosity patterns for the CD19 L174V polymorphism compared to European and East Asian populations that dominate genomic databases and CAR-T trials. Additionally, consanguinity-driven elevated homozygosity could amplify functional effects of homozygous variant carriage, making Arab cohorts uniquely informative for studying germline variation at this locus.

This study addresses this gap by characterizing CD19 variation in a large, publicly available Arab cohort, with the following objectives: (1) determining allele frequencies and genotype distributions for all non-synonymous CD19 variants, (2) comparing these frequencies to global reference populations, (3) assessing Hardy–Weinberg equilibrium deviation as a reflection of population structure, and (4) generating testable hypotheses on the potential impact of germline L174V homozygosity on CAR-T therapy outcomes.

## 2. Materials and Methods

### 2.1. Data Source and Cohort Description

We analyzed publicly available whole-exome sequencing data from individuals of Arab descent, predominantly Qatari nationals, accessed through the National Center for Biotechnology Information Sequence Read Archive (SRA) under accessions SRP060765, SRP061943, and SRP061463. These datasets were originally generated as part of Qatar Genome Program pilot studies and represent one of the largest publicly available genomic resources for an underrepresented population [19].

The final analytic cohort consisted of 1196 individuals. All included individuals were self-identified as having Arab family origins. The cohort was approximately balanced for gender (52% male, 48% female) with ages ranging from 18 to 85 years. Individuals with known malignancies, including hematological disorders, were excluded in these original studies, making this cohort representative of the general population for the purpose of hematological disorders. The original studies received ethical approval from the Qatar Biomedical Research Institute Institutional Review Board, and all participants provided written informed consent for genomic research and public data deposition.

### 2.2. Quality Control and Ancestry Inference

All samples underwent rigorous quality control prior to analysis. Variants were filtered to retain only those with read depth ≥ 30, genotype quality ≥ 30, and missingness < 5%. Relatedness analysis was conducted by the authors [19] using KING; however, for the purpose of this report, this is a limitation that we acknowledge. For downstream statistical an analysis, the final cohort comprises 1196 individuals.

Beginning with SAM files (imported from the Qatari Genome Project) as input, the workflow systematically removed group headers and filtered alignments by excluding unmapped reads, retaining only concordant (properly paired) read pairs, and selecting the identified unique mapping tags to eliminate ambiguous multi-mapping events. The filtered alignments were subsequently converted to BAM format using SAMtools (v1.21), sorted by genomic coordinates, and conditionally modified to add “chr” prefixes to chromosome identifiers for standardized genome reference compatibility, with SAMtools also employed for BAM indexing to enable efficient random access during variant calling.

The pipeline then completed the variant calling using the Pisces software (v5.2.11.163), which analyzes the curated high-quality alignments to generate a VCF file containing identified raw genomic variants. No post-calling filters were applied to the variants in the generated VCF file, as these represent germline variants rather than somatic mutations. Lastly, the annotation was performed using Variant Studio V3 to functionally characterize the identified variants and assess their potential biological significance [20].

### 2.3. Population Comparisons

To ensure comparability with reference databases, we applied identical quality filters to our data and to public data extracts: depth ≥ 30, genotype quality ≥ 30, and missingness < 5%. We acknowledge that differences in sequencing technology (Illumina HiSeq for our data vs. varied platforms for reference databases) and variant calling algorithms (Pisces v5.2 for our data vs. GATK for gnomAD) may introduce bias. Therefore, we present comparisons as descriptive enrichment ratios rather than statistically precise differences.

For population frequency comparisons, we utilized publicly available data from gnomAD v4.1 (genome aggregation database, containing 730,947 exomes and 76,215 genomes across global populations), the 1000 Genomes Project Phase 3 (2504 individuals from 26 populations), and ExAC (Exome Aggregation Consortium, 60,706 individuals) [16,21,22,23]. We specifically extracted allele frequencies for CD19 variants in the following super-populations: European (non-Finnish), African/African American, Latino/Admixed American, East Asian, South Asian, and other (including Middle Eastern where available).

### 2.4. Hardy–Weinberg Equilibrium and Inbreeding Analysis

Genotype frequencies for the CD19 variant (C/C, C/G, G/G) were tallied from observed counts. Allele frequencies were calculated by direct gene allele counts. Expected genotype counts under Hardy–Weinberg equilibrium (HWE) were derived using the standard equations: *E*_LL = *p*^2^ × *N*, *E*_LV = 2*pq* × *N*, and *E*_VV = *q*^2^ × *N*, where *p* is the frequency of the L (C) allele, q is the frequency of the V (G) allele, and N is the total number of individuals. A chi-square goodness-of-fit test with one degree of freedom was applied to compare observed and expected genotype counts, with statistical significance set at *p* ≤ 0.05.

To assess whether any observed deviation from HWE could be attributed to consanguinity (non-random mating), the inbreeding coefficient *F* was estimated as *F* = 1 − (*H*obs/*H*exp), where *H*obs is the observed heterozygosity and *H*exp = 2*pq* is the expected heterozygosity under HWE. Expected genotype counts under inbreeding were then recalculated using the adjusted equations: LL = *p*^2^ + *pqF*, LV = 2*pq* (1 − *F*), and VV = *q^2^* + *pqF*. For the R515H variant (rs34763945), 95% confidence intervals for allele frequencies were calculated using the Wilson score method and are provided in Table 1.

## 3. Results

Analysis of the *CD19* coding sequence in 1196 Arab individuals identified 21 non-synonymous variants (Supplementary Table S1) distributed across the extracellular domain, transmembrane domain, and cytoplasmic tail. Of these, 17 were missense variants and 4 were nonsense variants predicted to result in premature truncation. Notably, all variants except c.520C > G (L174V) were observed exclusively in the heterozygous state, with no homozygous alternate individuals identified for any of the remaining 20 variants (Supplementary Table S1). The median variant allele frequency across all non-synonymous variants was 0.8% (range 0.04% to 23.4% for the minor allele of rs2904880), consistent with the expected distribution of rare and common variation.

Two variants of potential clinical relevance were prioritized for detailed analysis based on their location within functional domains and prior literature implicating them in CAR-T resistance mechanisms. L174V (rs2904880) within the FMC63 scFv binding domain and R515H (rs34763945) within the transmembrane domain have been shown to affect CD19 surface expression and internalization dynamics [24,25].

### 3.1. The L174V Variant (rs2904880)

The L174V variant exhibited a V174 allele frequency of 76.6% in our Arab cohort; the minor allele (L174) frequency was 23.4%. Throughout this manuscript, L174 refers to the reference allele (leucine) and V174 refers to the variant allele (valine). This V174 allele frequency is notably higher than in European populations (68.6% in non-Finnish Europeans, representing a 1.11-fold enrichment) and comparable to frequencies observed in South Asian (83.2%) and African (94.0%) populations. Regarding reference allele designation: In gnomAD v4.1, the reference allele is C (encoding Leucine, L174); in 1000 Genomes Phase 3, the reference allele is also C; in ExAC, the reference allele is C. V174 (G allele) is consistently the alternate allele across all three databases.

The genotype distribution was: L/L (homozygous reference, leucine/leucine) = 144 individuals (12.0%); L/V (heterozygous) = 271 individuals (22.7%); and V/V (homozygous genotype, valine/valine) = 781 individuals (65.3%) (Table 1). The V/V rate of 65.3% attributed to homozygosity (GG) in our Arab cohort is 1.24-fold higher than in non-Finnish Europeans (52.5% homozygosity for V/V) and 1.80-fold higher than in Latino/Admixed American populations (36.3%) as reported by the 1000 Genomes Project [21].

Hardy–Weinberg equilibrium analysis for rs2904880 revealed highly significant deviation from expected genotype counts (*χ^2^* = 161.4, *df* = 1, *p* < 0.00001), characterized by a marked heterozygote deficit (observed 271 [22.66%] vs. expected 428.36 [35.82%]) and a reciprocal homozygote excess. This pattern is consistent with non-random mating. Estimation of the inbreeding coefficient yielded F = 0.367, suggests a high rate of consanguinity. After adjusting expected genotype counts using this F value, the observed counts were reproduced nearly exactly (Table 1, column 4). Despite the observed statistical significance, this HWE deviation remains not fully explained; the contribution of other factors such population structure, relatedness, and technical factors including duplicate samples cannot be ruled out, and that requires further computing verifications. The original data source (Qatar Genome Project) group applied the standard data quality checks of their own, however we cannot independently verify these at the current time. We further presented the inbreeding coefficient (*F* = 0.367) as a descriptive statistic rather than a definitive estimate of consanguinity.

### 3.2. The R515H Variant (rs34763945)

The R515H variant (rs34763945), located within the CD19 transmembrane domain, was observed at a MAF of 1.34% in our Arab cohort (Table 2).

This frequency is significantly lower than the European frequency of 6.7% and similar to frequencies observed in Amish populations (1.54%). The R515H substitution changes a positively charged arginine to a neutral histidine within the transmembrane domain, hypothetically affecting CD19 membrane orientation, homodimerization, or association with signaling partners such as CD21 and CD81 [26,27,28,29].

The observation that population-specific variation is bidirectional, with L174V more common in Arabs than Europeans but R515H less common, demonstrates that genetic differences at therapy-relevant loci cannot be predicted from global averages or from any single reference population. This underscores the necessity of direct empirical characterization of understudied populations rather than relying on imputation/extrapolation from centric databases alone.

### 3.3. Comparison with Recently Published Real-World Data

Hanbali et al. reported the first Middle Eastern real-world experience with tisagenlecleucel in 20 adolescent and young adult B-ALL patients from Saudi Arabia [30]. At 12-month median follow-up, 1-year RFS and OS were 56% and 74%, respectively; loss of B-cell aplasia occurred in 35% of patients at a median of 5 months, and 35% proceeded to allogeneic stem cell transplantation. Severe cytokine release syndrome (grade III–IV) occurred in 5% of patients [30], compared to reported background rates of 20–30 in other cohorts [31]. The Saudi study did not include CD19 genotyping, and therefore, the potential effect of the L174V polymorphism on outcomes remains unknown. Our genomic findings lay the foundation for future prospective studies integrating genotyping into CAR-T trials in Middle Eastern populations.

## 4. Discussion

This study provides the first comprehensive characterization of CD19 genetic variation in a large Arab cohort, addressing a critical gap in pharmacogenomic knowledge that has persisted despite the global expansion of CAR-T therapy. The principal finding of 76.6% V174 allele frequency (minor allele frequency of L174 = 23.4%) and 65.3% V/V homozygosity positions Arab populations at the upper end of the global spectrum for this potentially therapy-relevant variant. This finding generates testable hypotheses for future clinical investigations.

The location of L174V within the minimal recognition domain of the FMC63 scFv is particularly noteworthy. Structural studies have demonstrated that the V174 substitution induces conformational changes approximately 23 Å from the binding interface, potentially affecting binding kinetics through allosteric mechanisms rather than direct steric hindrance [25,27]. More compellingly, Zhang et al. demonstrated that FMC63 CAR-T cells effectively eliminated wild-type CD19-expressing cells but showed reduced killing of cells harboring CD19 mutations, while an alternative CAR (21D4) targeting a distinct membrane-proximal epitope retained efficacy against mutated targets [32].

A critical distinction is required here. These studies examined somatic mutations acquired during tumor evolution under therapeutic pressure, not germline polymorphisms present from birth in all B cells. The clinical significance of germline L174V homozygosity remains entirely unexplored. Our finding that 65% of this Arab cohort are V/V homozygotes compared to 52.5% of Europeans raises fundamental questions that remain unanswered pending functional and clinical studies.

### 4.1. Mechanistic Hypotheses and Testable Predictions

The following hypotheses are purely speculative and are not supported by experimental data from this study. They are presented solely as a framework to guide future research. We have not conducted binding assays, CD19 expression experiments, or structural modeling.

Based on the available structural and functional evidence, we propose three non-mutually exclusive mechanistic hypotheses regarding the potential impact of germline L174V status on CAR-T therapy outcomes. These hypothetical structural insights may partially explain the suboptimal clinical outcomes attributed to an altered scFv-CD19 interface, reflecting a subtle rather than complete CD19 epitope loss. Three testable hypotheses are therefore proposed as a framework for future investigations.

**Hypothesis** **1** (Binding Affinity Hypothesis)**.** *The V174 isoform might have reduced binding affinity for the FMC63 scFv compared to the L174 isoform. This hypothesis would predict that V/V genotypes could have higher thresholds for CAR-T activation, though the opposite effect is also possible. This hypothesis can be tested through surface plasmon resonance binding studies using recombinant CD19 isoforms and FMC63 scFv, as well as cellular avidity assays comparing CAR-T recognition of L174-expressing versus V174-expressing target cells [33,34].*

**Hypothesis** **2** (Epitope Accessibility Hypothesis)**.** *The substitution of valine which has a shorter side chain relative to leucine (reference residue) is predicted to weaken the CD19 hydrophobic packing against critical CDR residues, thereby exerting a modulatory (rather than absolute) effect on scFv-CD19 binding. The V174 substitution could potentially alter the conformational presentation of the FMC63 epitope. This could be tested using CD19 expression titration experiments and competition binding assays with conformation-specific antibodies [35].*

**Hypothesis** **3** (Antigen Escape Dynamics Hypothesis)**.** *Germline V174 homozygosity might alter the fitness landscape for somatic escape mutations. This can be tested through longitudinal sequencing of CD19 in patients relapsing after CAR-T therapy, stratifying by germline genotype [36,37].*

### 4.2. The Research Opportunity of Consanguineous Population Structure

Though statistically significant, the Hardy–Weinberg deviation may be partly explained by Arab demographic features: consanguinity rates of 25–60% and strong founder effects [17,38]. If biological in origin, elevated homozygosity in consanguineous populations could amplify any phenotype associated with homozygous variant carriage. If V174 homozygosity affects CAR-T outcomes, detection would be easier in Arab (65% homozygotes) than European (52% homozygotes) populations. Statistical power is substantially higher with greater homozygosity, especially for recessive/additive models [39].

### 4.3. Implications for Global Health Equity in Immunotherapy

Underrepresentation of Middle Eastern populations in CAR-T trials is both a missed scientific opportunity and an equity concern. As of 2025, <2% of enrolled participants in CD19 CAR-T trials are of Middle Eastern/North African ancestry, despite these regions comprising ~5% of the global population [40]. This disparity persists despite increasing regional availability: King Faisal Specialist Hospital has treated >200 patients with CAR-T and launched local manufacturing [https://www.kfshrc.edu.sa/en/news/2025/10/kfshrc-treats-200-patients-with-cart-therapy-and-launches-local-manufacturing, accessed on 1 June 2026], and King Hussein Cancer Center has started CAR-T trials with 17 enrolled patients (unpublished data). Arab populations exhibit distinct genetic profiles at a therapy-relevant locus. Whether these differences translate into differential outcomes remains unknown, as no binding, structural, or clinical data support such a claim. This is a potential source of health inequity if V174 homozygosity affects CAR-T efficacy or toxicity. Current trials have not included the population where this effect would be most detectable.

## 5. Limitations

First, our analysis uses public genomic data without clinical outcomes; no CAR-T trial has incorporated CD19 genotyping in this population. We cannot establish a direct link but provide a genomic foundation. Second, despite relatedness filtering (pi-hat > 0.125) and PCA, cryptic relatedness or substructure may persist; technical artifacts cannot be ruled out. Third, comparisons with reference databases are not harmonized due to differences in sequencing, variant calling, and coverage; we present descriptive comparisons. Fourth, while *n* = 1196 is substantial for an underrepresented population, it remains modest versus gnomAD; validation in independent Arab cohorts is needed. Fifth, functional studies of L174V’s effect on FMC63 binding kinetics are encouraged; we present no binding, expression, or clinical data.

## Figures and Tables

**Table 1:** Observed and expected genotype counts for CD19 L174V (rs2904880) under Hardy–Weinberg equilibrium (HWE) and after adjustment for consanguinity (inbreeding coefficient F = 0.367) ^4^.

Genotype	Observed (*n* [%]) ^1^	Expected Under HWE (*n* [%]) ^2,3^	Expected Under HWE + Consanguinity (*n* [%]) ^5^
L/L (C/C)	144 [12.04%]	65.32 [5.46%]	144 [12.04%]
L/V (C/G)	271 [22.66%]	428.36 [35.82%]	271 [22.66%]
V/V (G/G)	781 [65.30%]	702.32 [58.72%]	781 [65.30%]
Total	1196 [100%]	1196.00 [100%]	1196 [100%]

^1^ Allele frequencies: *p* = freq(L) = 0.2337; *q* = freq(V) = 0.7663. ^2^ HWE expectations: *E*_LL = *p*^2^*N*; *E*_LV = 2*pqN*; *E*_VV = *q*^2^*N*. ^3^ Chi-square test for HWE (df = 1): χ^2^ = 161.4; *p* < 0.00001. ^4^ Inbreeding coefficient: *F* = 1 − (*H*_obs/*H*_exp) = 0.3674. ^5^ Consanguinity-adjusted expectations: LL = *p*^2^ + *pqF*; LV = 2*pq*(1 − *F*); VV = *q*^2^ + *pqF*.

**Table 2:** rs34763945 allelic frequencies in the Arab cohort compared to other populations with 95% confidence intervals and sample denominators.

Genetic Ancestry Group	Allele Count	Allele Number	Sample Denominator (Individuals) *	Homozygotes	Allele Frequency (%)	95% CI (Lower)	95% CI (Upper)
European (non-Finnish)	78,926	1,179,742	589,871	2747	6.69%	6.65%	6.73%
European (Finnish)	4120	63,982	31,991	126	6.44%	6.25%	6.63%
South Asian	2084	91,074	45,537	45	2.29%	2.19%	2.39%
Admixed American	1327	59,990	29,995	13	2.21%	2.10%	2.33%
Ashkenazi Jewish	597	29,606	14,803	11	2.02%	1.86%	2.18%
Middle Eastern	117	6060	3030	5	1.93%	1.61%	2.31%
Amish	14	908	454	0	1.54%	0.92%	2.58%
African/African American	743	75,040	37,520	5	0.99%	0.92%	1.06%
East Asian	5	44,888	22,444	0	0.01%	0.01%	0.03%
**Our study**	**32**	**2392**	**1196**	**0**	**1.34%**	**0.95%**	**1.88%**

* Sample Denominator is the number of diploid individuals contributing to each estimate, calculated as the allele number divided by 2 (each individual carries two alleles at this locus).

## Data Availability

All sequencing data analyzed in this study are publicly available through the NCBI Sequence Read Archive under accessions SRP060765, SRP061943, and SRP061463. Analysis scripts and derived variant call files have been deposited at Zenodo (doi https://doi.org/10.1038/hgv.2016.16). The original data is publicly available [doi https://doi.org/10.1038/hgv.2016.16].

## Supplementary Materials

The following supporting information can be downloaded at: https://www.mdpi.com/article/10.3390/jpm16070343/s1, Table S1, title: (*CD19* coding variants identified in 1196 Arab individuals, ranked by alternate allele frequency).
