# Supplementary material for: Perspective for CAR T-Cell Therapy in Underrepresented Populations: A Hypothesis-Generating CD19 Genomic Analysis
Source: J Pers Med. 2026 Jun 25;16(7):343. doi: 10.3390/jpm16070343 (PMC13412835; doi:10.3390/jpm16070343)
Supplement: Supplementary file 1 [file jpm-16-00343-s001.zip › jpm-4274642-supplementary/jpm-4274642-supplementary.pdf]

Supplementary Table S1.

***CD19* coding variants identified in 1196 Arab individuals, ranked by alternate allele frequency**

HGVSc	Amino Acids	Protein Position	Allelic Frequency (%)	Transcript	Ref/Ref n (%)	Het n (%)	Alt/Alt n (%)
NM_001178098.1:c.520C>G	L/V	174	76.6	NM_001178098.1	144 (12.0%)	271 (22.7%)	781 (65.3%)
NM_001178098.1:c.1405A>C	T/P	469	2.8	NM_001178098.1	1129 (94.4%)	67 (5.6%)	0 (0.0%)
NM_001178098.1:c.1072A>C	T/P	358	1.9	NM_001178098.1	1151 (96.2%)	45 (3.8%)	0 (0.0%)
NM_001178098.1:c.1544G>A	R/H	515	1.4	NM_001178098.1	1163 (97.2%)	33 (2.8%)	0 (0.0%)
NM_001178098.1:c.875T>G	V/G	292	1.3	NM_001178098.1	1165 (97.4%)	31 (2.6%)	0 (0.0%)
NM_001178098.1:c.1460A>C	D/A	487	0.9	NM_001178098.1	1175 (98.2%)	21 (1.8%)	0 (0.0%)
NM_001178098.1:c.871A>G	K/E	291	0.9	NM_001178098.1	1174 (98.2%)	22 (1.8%)	0 (0.0%)
NM_001178098.1:c.1403T>G	L/R	468	0.6	NM_001178098.1	1181 (98.7%)	15 (1.3%)	0 (0.0%)
NM_001178098.1:c.1317C>A	Y/*	439	0.6	NM_001178098.1	1182 (98.8%)	14 (1.2%)	0 (0.0%)
NM_001178098.1:c.859A>G	T/A	287	0.5	NM_001178098.1	1185 (99.1%)	11 (0.9%)	0 (0.0%)
NM_001178098.1:c.1395T>G	D/E	465	0.4	NM_001178098.1	1187 (99.2%)	9 (0.8%)	0 (0.0%)
NM_001178098.1:c.1340T>C	L/P	447	0.4	NM_001178098.1	1186 (99.2%)	10 (0.8%)	0 (0.0%)
NM_001178098.1:c.1331A>G	D/G	444	0.4	NM_001178098.1	1186 (99.2%)	10 (0.8%)	0 (0.0%)
NM_001178098.1:c.189A>C	K/N	63	0.4	NM_001178098.1	1187 (99.2%)	9 (0.8%)	0 (0.0%)
NM_001178098.1:c.1522C>G	L/V	508	0.3	NM_001178098.1	1190 (99.5%)	6 (0.5%)	0 (0.0%)
NM_001178098.1:c.1383T>G	Y/*	461	0.2	NM_001178098.1	1191 (99.6%)	5 (0.4%)	0 (0.0%)
NM_001178098.1:c.1318G>C	E/Q	440	0.2	NM_001178098.1	1191 (99.6%)	5 (0.4%)	0 (0.0%)
NM_001178098.1:c.1396G>A	E/K	466	0.1	NM_001178098.1	1193 (99.7%)	3 (0.3%)	0 (0.0%)
NM_001178098.1:c.1379C>T	S/F	460	0.1	NM_001178098.1	1193 (99.7%)	3 (0.3%)	0 (0.0%)
NM_001178098.1:c.886A>G	T/A	296	0.1	NM_001178098.1	1193 (99.7%)	3 (0.3%)	0 (0.0%)
NM_001178098.1:c.775T>G	Y/D	259	0.1	NM_001178098.1	1193 (99.7%)	3 (0.3%)	0 (0.0%)

Coding-region variants of *CD19* (transcript NM_001178098.1) identified in the Arab cohort (*n* = 1196), listed in order of decreasing alternate allele frequency. Each row reports the HGVS coding-DNA description (HGVSc), the encoded amino-acid change in one-letter code (* denotes a stop-gained/nonsense substitution), the affected protein position, the alternate allele frequency (%), and the genotype distribution; reference homozygotes (Ref/Ref), heterozygotes (Het), and alternate homozygotes (Alt/Alt), given as counts and percentages of the 1196 genotyped individuals.
